# A Brief History of Giant Viruses’ Studies in Brazilian Biomes

**DOI:** 10.3390/v14020191

**Published:** 2022-01-19

**Authors:** Paulo Victor M. Boratto, Mateus Sá M. Serafim, Amanda Stéphanie A. Witt, Ana Paula C. Crispim, Bruna Luiza de Azevedo, Gabriel Augusto P. de Souza, Isabella Luiza M. de Aquino, Talita B. Machado, Victória F. Queiroz, Rodrigo A. L. Rodrigues, Ivan Bergier, Juliana Reis Cortines, Savio Torres de Farias, Raíssa Nunes dos Santos, Fabrício Souza Campos, Ana Cláudia Franco, Jônatas S. Abrahão

**Affiliations:** 1 Laboratório de Vírus, Departamento de Microbiologia, Universidade Federal de Minas Gerais, Belo Horizonte 31270-901, Minas Gerais, Brazil; pvboratto@gmail.com (P.V.M.B.); mateusmserafim@gmail.com (M.S.M.S.); asawitt1997@gmail.com (A.S.A.W.); anapbio2@gmail.com (A.P.C.C.); azvdobruna@gmail.com (B.L.d.A.); neogaps@gmail.com (G.A.P.d.S.); isabellaaquino92@gmail.com (I.L.M.d.A.); bastostalita04@gmail.com (T.B.M.); victoriafq18@gmail.com (V.F.Q.); rodriguesral07@gmail.com (R.A.L.R.); 2 Embrapa Pantanal, Corumbá 79320-900, Mato Grosso do Sul, Brazil; bergiercpap@gmail.com; 3 Departamento de Virologia, Instituto de Microbiologia Paulo de Góes, Universidade Federal do Rio de Janeiro, Rio de Janeiro 21941-590, Rio de Janeiro, Brazil; cortines@micro.ufrj.br; 4 Laboratório de Genética Evolutiva Paulo Leminsk, Departamento de Biologia Molecular, Universidade Federal da Paraíba, João Pessoa 58050-085, Paraíba, Brazil; stfarias@yahoo.com.br; 5 Laboratório de Virologia, Departamento de Microbiologia, Imunologia e Parasitologia, Instituto de Ciências Básicas da Saúde, Universidade Federal do Rio Grande do Sul, Porto Alegre 90.050-170, Rio Grande do Sul, Brazil; engraissanunes@gmail.com (R.N.d.S.); camposvet@gmail.com (F.S.C.); anafranco.ufrgs@gmail.com (A.C.F.)

**Keywords:** amoebae viruses, Brazilian isolates, giant virus, NCLDV, virosphere, virus diversity

## Abstract

Almost two decades after the isolation of the first amoebal giant viruses, indubitably the discovery of these entities has deeply affected the current scientific knowledge on the virosphere. Much has been uncovered since then: viruses can now acknowledge complex genomes and huge particle sizes, integrating remarkable evolutionary relationships that date as early as the emergence of life on the planet. This year, a decade has passed since the first studies on giant viruses in the Brazilian territory, and since then biomes of rare beauty and biodiversity (Amazon, Atlantic forest, Pantanal wetlands, Cerrado savannas) have been explored in the search for giant viruses. From those unique biomes, novel viral entities were found, revealing never before seen genomes and virion structures. To celebrate this, here we bring together the context, inspirations, and the major contributions of independent Brazilian research groups to summarize the accumulated knowledge about the diversity and the exceptionality of some of the giant viruses found in Brazil.

## 1. Introduction

The description and characterization of the first amoebal giant virus (GV) in 2003, Acanthamoeba polyphaga mimivirus (APMV), raised important questions regarding the limits of the virosphere. These first findings revealed viral particles of about 700 nm, non-filterable through 0.2 µm pore size filters [1]. Although not the first described nucleocytoplasmic large DNA virus (NCLDV) (phylum *Nucleocytoviricota*), which includes other families such as *Poxviridae* [2], the original discovered member of the family *Mimiviridae* motivated new interpretations of crucial features in an organism recognized as a virus, advancing both knowledge of, and perspectives on, the most abundant group of organisms on Earth [3].

Much of the subsequent work on GVs has been driven by curiosity and the possibility of isolating novel groups of amoebal viruses and finding intriguing new characteristics. For instance, in 2008 La Scola et al. had previously isolated a distinct strain of APMV, the acanthamoeba castellanii mamavirus, together with the first ever described virophage, the Sputnik virus (SNV), both found in a water-cooling tower of a hospital in France [4]. Here, the interest in describing more GVs contributed by influencing the consolidation of “virophages” as new satellite-like viruses, which are dependent on the mimivirus factory for their replication by putatively hijacking some key features (e.g., the viral RNA polymerase) [5].

Similarly, in 2009, Boyer et al. reported the isolation of the marseillevirus, a novel GV for which analysis of its core genes suggested a previously uncharacterized family of NCLDV. In addition, by unveiling some of the main features of the genome’s repertoire for this new GV, the authors have proposed amoebas as potential “melting pots” of microbial evolution, given the convenient intracellular environment for gene transfer among parasites, including complex genomes that could advent from different GVs’ viral sources [6]. Some of the GVs’ hosts (different amoeba genus, e.g., *Acanthamoeba*) are indeed considered ubiquitous, found in almost all latitudes [7,8], as well as in a wide-range of environments, including wastewater [9], terrestrial and (deep) marine water [7,10], thermal springs [11], permafrost [12], ventilation and air conditioning systems, and even in hospital settings [13,14]. Notwithstanding, GVs can be found in a large set of different native hosts or host-associated organisms, from other various species of amoebas [8,15] to filtering feeding organisms such as oysters [16]. Recently, metagenomics studies have also indicated that GVs are even more abundant in marine environments than prokaryotes, suggesting that these viruses may play a fundamental role in nature as biological control agents, regulating biogeochemical cycles, and potentially acting as evolutionary driving forces [17,18,19]. Ultimately, hijacking or utilizing cellular components and translational machinery may indicate a common origin, regarding information on life’s evolution, and the presence of translation proteins may open new hypotheses about GVs’ origin and phylogenetic relationships with other domains of life [20].

**Figure 1:**
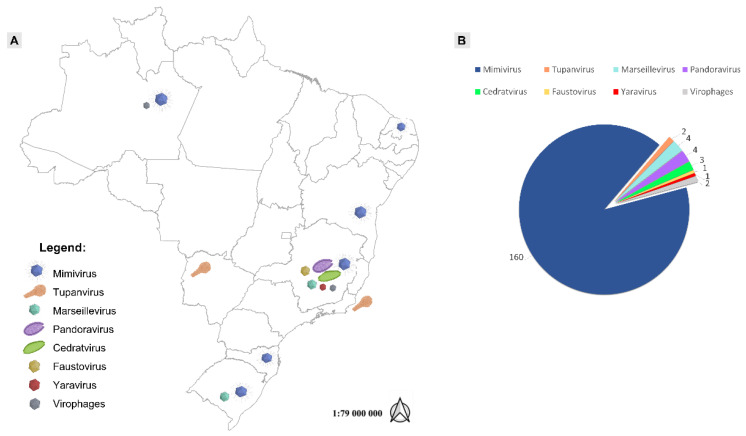
Location and numbers of giant viruses isolated in Brazil. (**A**) Schematic map showing the sites of isolation for the major groups of giant viruses discovered in Brazil. (**B**) Number of isolates discovered for each of these groups.

The broad-spectrum environmental profile of GVs made Brazil an interesting field to search and study these microorganisms, especially considering the wide range and diversity of environments and biological dispersion throughout biomes, settings and native habitats around the country, such as the Amazon forest and Cerrado savannas, as well as the Pantanal wetlands, including the soda lakes of Nhecolândia in the middle of the woods, which hide rich organic sediments [7,10,21,22,23]. These characteristics were reflected in the findings and discoveries of several GV isolates in the Brazilian territory, such as: (i) tupanvirus soda lake, isolated from the Brazilian Pantanal (Nhecolândia lakes) and tupanvirus deep ocean, isolated from sediment at 3000 m below the water surface line at ‘Bacia de Campos’, in Rio de Janeiro [10]; (ii) samba virus (SMBV), isolated from the Brazilian Amazon [22]; (iii) cedratvirus getuliensis, from sewage samples in Minas Gerais state [24]; (iv) niemeyer virus [25], faustovirus mariensis [26] and yaravirus [27], all of them isolated from the Pampulha urban lagoon; (v) a number of *Mimivirus* isolates [7,21]; and many others (Figure 1A,B), some of which we will further discuss in this review.

## 2. Giant Viruses Discovery and Isolation

### 2.1. Mimiviruses Boosted Amoebal Giant Viruses’ Research

The first amoebal GV isolated in Brazil dates from 2011. During a field trip to the Brazilian Amazon, aiming to search for GVs, water samples were collected from the Negro River, in Manaus city. These samples were then assessed for prospection using *Acanthamoeba castellanii* cells as an isolation platform, which allowed the discovery of the first Brazilian GV, named samba virus (SMBV) [22].

Samba viruses have mimivirus-like particles, showing capsids with an average diameter of 527 nm, surrounded by fibrils of 155 nm [28]. The SMBV genome is composed of about 1.2 Mb, and the phylogenetic analysis clustered it within the lineage A of the mimiviruses (Table 1). Analysis of the SMBV replication cycle using a set of electron microscopy images showed several similarities with the APMV replication cycle. Moreover, these images also revealed the presence of smaller viral particles that were further confirmed as the first Brazilian virophage named Rio Negro virophage (RNV) [22]. A few years later, RNV genome was sequenced and assembled presenting 18,145 bp, very similar to the sputnik 2 virophage genome [29].

Later, a new sputnik-like virophage named guarani virophage was also isolated from water samples obtained in the Pampulha Lagoon, Belo Horizonte city, Minas Gerais state. A deep characterization of its genome (18,967 bp) was performed, and its replication is described as rather slow (replication at 4 h.p.i. and particles morphogenesis at 16 h.p.i.) when compared to the cycle of its associated GV [30].

After SMBV discovery, several mimiviruses belonging to the three currently known lineages (A, B and C) were isolated from different Brazilian environmental samples, such as the so-called “Br-mimiC”, mimivirus golden (MVGD), isolated from golden mussels (*Limnoperna fortunei*) from Guaíba Lake, Rio Grande do Sul, in 2014 [31] and mimivirus gilmour (MVGM), isolated from water collected at the Pampulha Lagoon, in 2015 [21]. In this same work, the isolation of another 64 mimiviruses were described from water samples collected at the Pampulha Lagoon. They were obtained from three different *Acanthamoeba* species (*A. castellanii*, *A. polyphaga* and *A. griffini*), and had representatives in the three lineages of mimiviruses [21]. Also in 2015, 20 mimiviruses belonging to the lineage A were obtained from oyster-related samples of three different coastal regions of Brazil [16]. Considering their water-filtering capacity, these bivalves were tagged as excellent sources for the isolation of new GVs because their body allows the accumulation of both viruses and amoebas [16]. 

**Figure 2:**
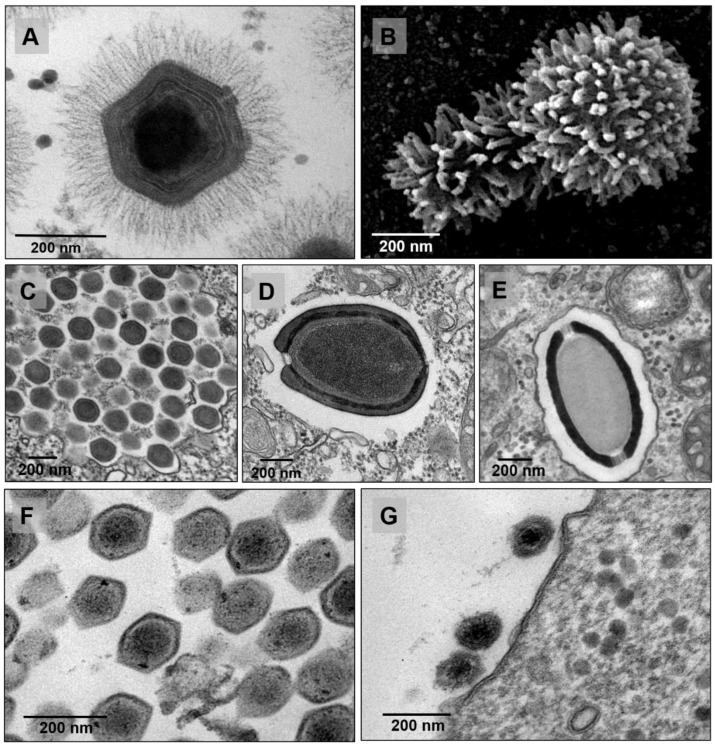
Panel with TEM images for the major groups of amoebal viruses isolated in Brazil. (**A**) mimivirus, (**B**) tupanvirus (source: 10.1038/s41598-018-36552-4), (**C**) marseillevirus, (**D**) pandoravirus, (**E**) cedratvirus, (**F**) faustovirus, and (**G**) yaravirus.

In another study, a mimivirus-related isolate called Niemeyer virus (NYMV) was discovered, once again from water samples obtained from the Pampulha Lagoon [25]. NYMV has a genome of approximately 1,299,140 bp, harboring a set of duplicated aminoacyl-tRNA synthetases, which suggests that such duplications may be important for the evolutionary history of mimiviruses (Table 1). In 2017, another lineage A mimivirus was described, this time from water samples collected from an urban lake at the Lagoa Santa city, also in the Minas Gerais state, and it was named kroon virus (KV) (Figure 2A) [32]. The study of KV (1,221,932 bp genome [33]) has established an interesting view of the distinct ways by which the major capsid protein (MCP) mRNA can be differentially processed, depending on the lineages of mimiviruses (Table 1) [32]. Apparently, for each of these mimiviruses’ lineages there is a genetic layout concerning how the MCP gene is organized in terms of its exons/introns and how they are arranged. As an example, in the KV study the nucleotide sequences of the third exon of the MCP (observed in the genome of all mimi-viruses) was described to be an alternative marker to disentangle each of the three lineages. In addition, a different form of mature mRNA was also described in transcripts of the MCP for mimiviruses of a given lineage (e.g., APMV and KV) [32]. Subsequently, in 2018, 64 giant viruses of the *Mimiviridae* family (26 from lineage A, 13 from lineage B, two from lineage C and 23 from unidentified lineages) were described from various types of samples, including marine water from Antarctica, which was the first time to our knowledge that mimiviruses were isolated in this continent [7].

The year 2018 has also been marked by the description of one of the longest and most complex viruses described to date, obtained from a set of samples collected from extreme aquatic environments. The tupanvirus soda lake was isolated from samples collected from an alkaline salty lake (Nhecolândia, Pantanal, Brazil) while the deep ocean tupanvirus was obtained from 3000 m depth sediment samples below the Atlantic Ocean at ‘Campos dos Goytacazes’ (Figure 2B) [10]. In contrast to other giant viruses previously isolated, these GVs are able to infect and establish a productive cycle in many species of amoebas, including *Acanthamoeba* spp., *Vermamoeba vermiformis*, *Dictyostelium discoideum* and *Willartia magna* [10]. The tupanviruses’ capsid is similar to that of other mimiviruses already described in this review, around 450 nm in diameter, with a pseudo-icosahedral symmetry, covered by a layer of fibrils. They also present a “stargate” vertex, that is, a noticeable star-shaped opening at one capsid vertex. Interestingly, however, these viruses have a tail attached to the capsid, which is also covered with fibrils, a feature never seen before for amoebal viruses (Figure 2B). Due to the plasticity of this tail, the particles can vary from 1.2–2.3 µm in length, making it the longest virus ever described [10].

The genomes of the tupanviruses are complex and composed of double-stranded DNA of 1.44–1.51 Mb, encoding 1276–1425 predicted proteins (Table 1) [10]. Phylogenetic analysis using the DNA polymerase B family gene and other unique features exhibited by these viruses suggested that the tupanviruses group together with other mimiviruses form a distinct clade, which supported the proposal to form a new genus called “*Tupanvirus*” [10,34]. These viruses have been shown to be even more surprising, as deep genome analysis detected the largest translational apparatus ever described in the virosphere, with 20 aminoacyl-tRNA synthetases (aaRS), 67–70 tRNAs, in addition to other proteins in the translation process, such as translation factors (initiation, elongation and release) and proteins related to tRNA and mRNA maturation [10,34,35]. In addition, 20% of their genome is similar to genes originating from cellular organisms, with 9% from eukaryotes (of these, 3% originate from amoebas), 3% from archaea and 8% from bacteria [35].

These findings support data that demonstrate how other groups of organisms are relevant in studying the evolution of NCLDV genomes (Table 1). The fact that they have these genes shared with members of other cellular domains suggests that tupanviruses could also be found in non-extreme environments [35]. Altogether, the genetic arsenal of these and other mimiviruses within the virosphere add new levels of complexity to the understanding of the tree (or rhizome [36]) of life [20,37].

### 2.2. The Second Family Arises: The Discovery of Marseilleviruses

After the discovery of the first mimiviruses, the search for GVs intensified. In 2009, a virus named *Marseillevirus marseillevirus* was isolated in a biofilm from a water cooling tower in Paris, France [6], which gave rise to the family *Marseilleviridae*, officially recognized by the International Committee on Taxonomy of Viruses (ICTV) in 2013 (Figure 2C) [38]. Since then, other marseilleviruses have been isolated from different sources: (i) Lausannevirus, was discovered in water samples collected from the Seine river, in 2011 [39]; (ii) Cannes 8 virus was isolated from water in a cooling tower in Cannes, in 2013 [40]; (iii) tunisvirus and Fontaine Saint-Charles virus were isolated from freshwater collected in decorative fountains in Ariana, Tunisia, and in France, respectively [41,42]; (iv) insectomime virus was isolated from the internal organs and digestive tract of a dipteran drone fly’s larvae [43]; (v) Senegalvirus was discovered during metagenomic analysis of the bacterial diversity in the human gut microbiota from a apparently healthy African individual, in 2012 [44,45]; (vi) In 2014, the genomic characterization of Melbournevirus was reported, isolated from a freshwater pond in Melbourne, Australia [46]; and (vii) Port-Miou virus, isolated from a sample from a brackish submarine spring, in the Cassis Port-Miou Calanque, France, in 2015 [47].

Furthermore, different phylogenetic lineages of marseillevirus have been described. Initially, the phylogenetic analysis suggested the existence of three distinct lineages: Lineage A, consisting of *Marseillevirus*, Cannes 8 virus, Senegalvirus and Melbournevirus; Lineage B, consisting solely of Lausannevirus; and Lineage C, consisting of tunisvirus and insectomime virus [42]. That was based on phylogenetic reconstructions carried out with core genes including the NA polymerase B family, the VV A18 helicase, the D5 primase–helicase, the very late transcription factor 2B and the MCP [42].

**Figure 3:**
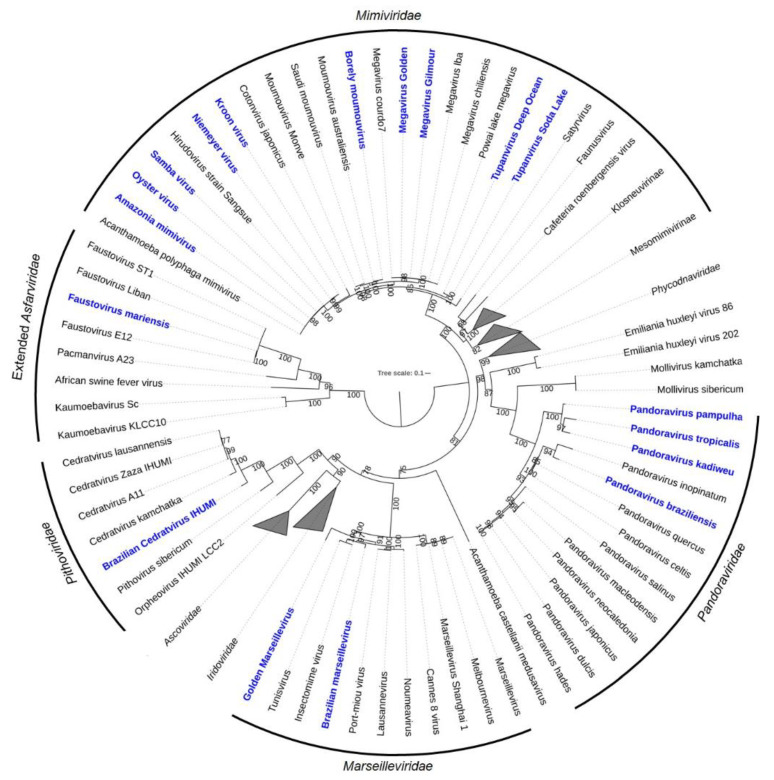
Maximum likelihood phylogenetic tree based on amino acid sequences of DNA polymerase B family of *Nucleocytoviricota*. Brazilian isolates are bold and highlighted in blue. Sequences were aligned using Muscle [48] and low conserved regions were removed using trimAl [49]. The tree was built using IQ-TREE [50] with 1000 ultrafast bootstrap replicates and the VT+F+R7 model chosen by ModelTest according to Bayesian Information Criterion. The tree was visualized in iToL [51]. The tree scale indicates the substitution rate.

However, the discovery of the first marseillevirus in America resulted in the creation of a new lineage in the family. The Brazilian Marseillevirus (BrMV) was described in 2014 from a sewage sample from a treatment station in the Pampulha lagoon [47]. The new lineage is supported by comparative genomic analyses highlighting several divergences between BrMV and other marseilleviruses (Figure 3) [47].

A few years later, in 2016, the golden marseillevirus (GMar) was described as a new member isolated from golden mussels collected in southern Brazil [52]. The structure of the virus particles strongly resembled other marseilleviruses, with particles of approximately 200 nm, obtained from a coculture with A. polyphaga. The genome is composed of a circular dsDNA with 360,610 bp, comparable in size to the genomes of other members of the family *Marseilleviridae*, which range from 346,754 bp to 386,631 bp for lausannevirus and insectomime virus, respectively (Table 1) [52]. A total of 483 ORFs were characterized. Curiously, despite this genome size similarity, the GMar genes’ content harbors 48.03% uncharacterized proteins. Many of these uncharacterized proteins can be considered as orphan genes (ORFans), also reported in other GVs such as Pandoravirus with 93% ORFans [53]. In addition, comparatively to the 212 genes shared among Brazilian marseillevirus, marseillevirus, lausannevirus, tunisvirus and golden marseillevirus, there are fourteen non-shared genes, of which seven are among the GMar genes [52].

### 2.3. Opening the GVs’ Box: The Discovery of Pandoraviruses

Ten years after the discovery of the first GVs, the description of a brand-new group of amoebal viruses has led virologists to become again surprised, as a series of new paradigms started to be challenged and the study of modern virology advanced. At the time, the discovery and characterization of the pandoraviruses established for the first-time a group with viral particles with sizes as great as 1 µm in length and genomes that exceeded the mark of 2.5 Mb, with an astonishing number of 93% of genes without recognizable homologs in available databases (e.g., GenBank) (Figure 2D) [53].

Before the investigations in Brazil, it is important to mention the initial studies regarding the first representatives of this group. Starting from 2013, these discoveries were made: (i) *Pandoravirus salinus*, isolated from a superficial sediment layer collected at the mouth of the Tunquen river in Chile; and (ii) *Pandoravirus dulcis*, isolated from a mud taken at the bottom of a freshwater pond near Melbourne, Australia [53]. A couple of years later, a study led to the reinvestigation of an endosymbiont isolated from an *Acanthamoeba* strain and concluded, by whole genome sequencing, that this organism was in fact a pandoravirus isolate, named Pandoravirus inopinatum [54,55]. In another work, a newly characterized isolate called Pandoravirus celtis was used to investigate a putative scenario in which the genetic divergence among the different isolates of pandoraviruses was caused by an ability of these viruses to perform the creation of genes through a de novo microevolution process [56].

During the years 2018 to 2019, two different studies carried out a series of in-depth approaches focusing on establishing a detailed view of the diversity of pandoraviruses, their evolution processes and aspects of their replication cycle [57,58]. In the first study, three samples of pandoravirus were first isolated and named as pandoravirus quercus, pandoravirus neocaledonia and pandoravirus macleodensis. Their replication cycles were independently investigated and interestingly, for the first time, the mature particles of pandoraviruses were filmed while being exocytosed by vesicles which were full of viruses [57]. The genomes of these isolates were fully sequenced and a new stringent reannotation protocol was established. With this new methodology, the genetic analysis of different isolates suggested a still open pan-genome for GVs, in which each novel isolate is predicted to be responsible for contributing more than 50 additional genes [57].

For the second study, three novel Brazilian isolates were used: (i) pandoravirus kadiweu, coming from samples of water collected in the city of Bonito, Mato Grosso do Sul; (ii) pandoravirus pampulha, and (iii) pandoravirus tropicalis, both coming from samples of water from an artificial lake located at the city of Belo Horizonte, Minas Gerais [58]. Here, the microscopy analysis was an important tool, not only to reinforce some already established data but also to reveal new features of the virus replication. As for other GVs, within 30 min of infection the pandoravirus virions were phagocytosed and engulfed inside a host vesicle called the phagosome [57]. This structure quickly fuses with lysosome-like organelles and triggers the next stage of replication, which is the start of viral uncoating [53,57]. The next step involves an intense manipulation of the host cell and deep modification of the cytoplasm environment in order to make the region of viral morphogenesis, known as the viral factory. The loss of the cell nucleus and an intense recruitment of the host membranes and mitochondria are necessary for this. The beginning of viral morphogenesis does not seem to have a polarization, as thought earlier [53]. Finally, the viral cycle ends with the host cell lysis [53,57].

Interestingly, however, it was observed in some microscopy images that several pandoravirus particles were packaged inside vesicles and transported to the periphery of the host cell before amoebal disintegration. Additionally, one-step-growth curves have shown the beginning of viral release around 6 to 9 h post-infection, before the onset of the amoebas’ lysis [58]. These results, together with data that show a negative impact on pandoravirus release by cells treated with brefeldin (a membrane traffic inhibitor), suggest an important role of exocytosis for early liberation of pandoravirus particles in an amoeba infection [58]. Such observations are commonplace to other GVs with analogous replication cycles, including, for example, the cedratviruses described in the next section.

### 2.4. A Double-Corked GV: Isolation and Characterization of the Cedratviruses

Viruses belonging to the cedratvirus group were first detected in 2016, with the isolation of Cedratvirus A11, a viral representative coming from diverse environmental samples collected in Algeria [59]. Their structure is constructed by a ~1 µm ovoid-shaped particle, resembling some morphological features of the pithovirus virions, though with a notable difference: the presence of two corked regions (instead of a single one) at the extremities of the particle [59]. Their genome is composed of a circular dsDNA with about 590 kbp, and it has been found to share a close relationship with the genomes of the two currently known pithoviruses (both in size and in genome content), pithovirus sibericum and pithovirus massiliensis [59].

The second cedratvirus isolate, called cedratvirus lausannensis, was obtained in an attempt to look for amoebal-resisting bacteria inside a drinking water plant located at the Morsang-sur-Seine commune, in France [60]. Four other isolates have been discovered since then: (i) cedratvirus zaza IHUMI, deriving from samples of sterile distilled water collected near Toulon city, in France; (ii) Brazilian cedratvirus IHUMI, collected from water samples supplemented with bio-floc in Belo Horizonte city, Brazil; (iii) cedratvirus Kamchatka, obtained from a muddy grit soil collected next to a volcano area in Russia; and (iv) cedratvirus getuliensis (Figure 2E), collected from sewage samples from the Itaúna city, Brazil [24,61,62]. Interestingly, the isolate Brazilian cedratvirus IHUMI is a representative of the group which harbors both particle and genome sizes with remarkable differences in comparison with the other cedratviruses discovered so far. The virion is approximately 910 nm in length, with some of the particles reaching around 696 nm, and the genome is also smaller, with a DNA molecule of 460,038 bp (Table 1) [63]. Comparative genomic analysis also indicated that this Brazilian isolate is the founding member of a new lineage of cedratviruses (Figure 3) [63].

In 2018, through the analysis of a series of electron microscopy images and by performing biological assays, an interesting study has helped to reveal most of the steps in the replication of cedratviruses. As expected for an amoebal virus with large-sized virions, the viral cycle starts by the particles getting into the infected cell through the exploration of a phagocytic pathway that is physiologically presented by the host [24]. Corroborating this observation, lower titers of the cedratvirus virions are observed when the infected amoebal cells are pre-treated with cytochalasin D, an inhibitor of phagocytosis. The cycle then progresses to the formation of an electron-lucent viral factory (as large as the cellular nucleus) in the cytoplasm of the infected amoeba and, differently from observed during infection of most giant viruses, the cellular nucleus seems to remain intact [24].

However, some typical cellular alterations are still observed, such as the recruitment of mitochondria around the viral factory region, the polarization of lysosomal vesicles in the infected cell and an intense traffic of membranes which were seen to be important during the morphogenesis of cedratvirus virions [24]. This step is described as very complex and relies on the formation of several membrane precursors (or crescents) which later assume the correct conformation of a mature viral particle. Finally, the viral cycle ends with the mature particles released via cell lysis or exocytosis [24]. Cedratviruses also present structural similarities and infection features to other GVs, such as the orpheoviruses, as discussed below.

### 2.5. Another Amoeba, Another Virus: Discovery and Characterization of Orpheovirus

By implementing amoebas of the *Vermamoeba vermiformis* species as a platform of isolation, new groups of viruses were discovered from different samples. Among them, an Orpheovirus was isolated in Marseille, France, from samples of rat stool [62]. Nevertheless, Souza et al. observed that, differently to previous findings of viruses infecting amoeba, CPE caused by Orpheovirus could be split into an early stage (3 to 12 h.p.i.), when cells stretch into a branched fusiform shape, and a late stage (starting at 24 h.p.i.), when cells become rounded [64].

The in-depth characterization of the replication cycle demonstrates that it takes approximately 30 h to be completed. It is suggested that one or more particles of Orpheovirus, which are around 1.1 μm, are phagocytized by the host cell within 1 h.p.i. [62,64]. After entry, the particle’s internal content is released when the membrane that surrounds the viral core fuses with the endosomal membrane through a structure called ostiole, located at the apex of the particle. Subsequently, the formation of the large electron lucent viral factory is observed, concomitantly with the recruitment of mitochondria and membranes [64]. Membrane recruitment and bleb formation also seems to be important for the viral factory formation and particle morphogenesis since they are affected by treatment with a membrane trafficking inhibitor at the middle stage of infection (8 h.p.i.), which is also observed for cedratviruses [24]. Similarly, as described for other viruses, the particle morphogenesis initiates with the formation of electron-dense semicircular structures, which are filled with their internal content until the formation of the complete closed particle [64].

The complete particle presents smaller fibrils, when compared to mimiviruses, and at least two layers between the fibril layer and the inner membrane [62,64]. Finally, the infectious particles start to be released by exocytosis, detected in the supernatant at 12 h.p.i. Moreover, it is observed that cell lysis also plays a role in viral particle release, mostly at late timepoints of infection. Along with the infectious particles, the formation of defective particles is also observed [64].

### 2.6. The Isolation and Characterization of Faustoviruses

The faustoviruses are a group of giant viruses first detected in 2015 from samples of sewage from different regions in France and in Dakar, Senegal [65]. In Brazil, the first representative of this group was isolated and described in 2019, from prospecting studies of water samples from the Pampulha lagoon. Faustovirus mariensis, as it was called, is a virus with icosahedral particles reaching approximately 190 nm in diameter and inducing cytopathic effects on amoebas of the *Vermamoeba vermiformis* species (Figure 2F) [26]. Their genome is composed of a circular, double-stranded DNA molecule of about 466,080 bp (Table 1). Like other GVs, the f. mariensis replication cycle starts with the infection of the amoeba in its trophozoite form. This infection progresses to the formation of a large electron-lucent viral factory and the recruitment of mitochondria to its periphery [26]. The morphogenesis of f. mariensis is similar to that of other faustoviruses previously described in the literature, with new mature particles being formed in small honeycomb structures within the cytoplasm of the host cell. Lysis of the infected cell is the most important means of releasing the f. mariensis progeny described so far [26].

In a rare antiviral strategy described for GVs and their amoebal hosts, Borges et al. have observed that the infection of *Vermamoeba vermiformis* cultures is able to trigger a process of encystation of the neighboring cells, trapping the particles of f. mariensis inside their host and preventing further infection in the population of amoebas [26]. This event, considered to be observed for the first time in these viruses, was directly influenced by f. mariensis infection at a multiplicity of infection (MOI) dependent rates. When cysts were derived from cells infected at high MOIs, they were permanently incapable of excysting, therefore becoming trapped inside the particles of f. mariensis. However, when these amoebal cells came from infections at lower MOIs, only the cells with neither viral particles nor factories were able of excysting [26].

Faustoviruses are also phylogenetically related to kaumoebaviruses and asfarviruses, with the hypothesis that a common ancestor is shared between these viruses (Figure 3) [62]. After analysis considering this evolutionary proximity, motifs that play the role of promoter sequence in asfarvirus have been identified within the faustovirus genome, leading to the conclusion that rich A-T (TATTT and TATATA) regions may also have an important role in the gene expression of both kaumoebavirus and faustovirus. These findings shed new light for a better understanding of giant virus’s gene expression [66]. As aforementioned, intriguing information regarding the GVs’ discovery and characteristics are quite common, and some unique factors have attracted attention in the field, such as the recent discovery of the yaravirus in 2020 [27].

### 2.7. Yaravirus, a Small Virus among the Giants

**Figure 4:**
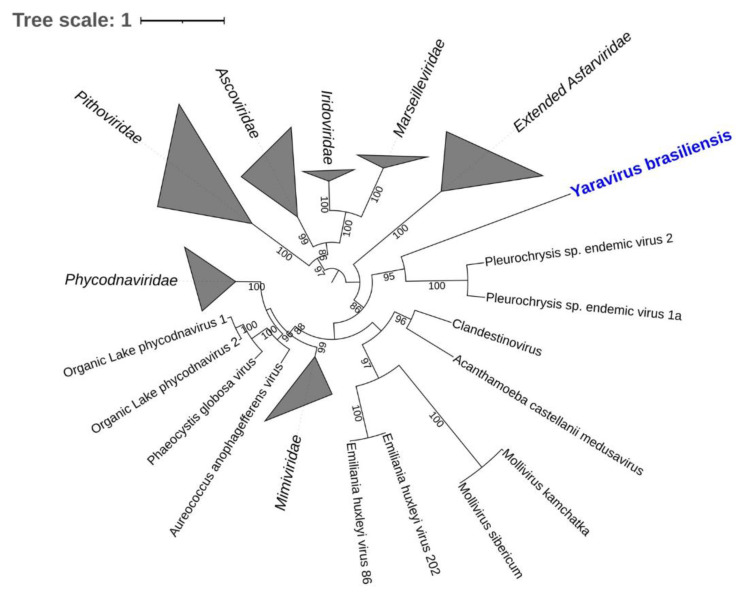
Maximum likelihood phylogenetic tree based on amino acid sequences of major capsid protein of Nucleocytoviricota. Yaravirus brasiliensis is bold and highlighted in blue. Sequences were aligned using Muscle [48] and low conserved regions were removed using trimAl [49]. The tree was built using IQ-TREE [50] with 1000 ultrafast bootstrap replicates and the VT+R3 model chosen by ModelTest according to Bayesian Information Criterion. The tree was visualized in iToL [51]. The tree scale indicates the substitution rate.

In late 2020, the discovery of a new lineage of dsDNA virus would enhance our knowledge on the diversity and evolution of viruses in amoeba. The yaravirus brasiliensis, as it was called, has been described as a novel virus of *Acanthamoeba castellanii,* harboring a genome of ~45 kbp enclosed in an icosahedral particle of about 80 nm in diameter [27]. Differently from any other virus isolated from acanthamoeba so far, this virus does not seem to share many of the features which are thought to represent the NCLDV, as it has neither a large particle nor a complex genome (Figure 4) [27]. This may indicate one of the following: (i) yaraviruses either belong to an extremely reduced group of amoebal viruses which are part of the NCLDVs; or (ii) these viruses represent the first discovered lineage of amoebal viruses that are not part of this complex group [27].

The genome of yaraviruses is composed mainly of ORFans, with an astonishing percentage of ~90% of their genes with functions never described before [27]. The search for yaravirus sequences in a huge dataset consisting of more than 8500 publicly available metagenomes from the most diverse habitats around the globe has also shown hits with distant homologs for the ATPase gene (NCVOG0249), with amino acid similarities that represented a number lower than 33% [27]. The discovery of yaravirus demonstrates how important are studies focusing on isolating new viruses from the environment [27]. Although metagenomics analyses have an important role in describing new viral species by using their standard methods [67,68,69], microorganisms like yaraviruses would be very difficult to discover, as these protocols mostly involve recognition of genes already described [27]. This finding could also be seen as a marking point to revamp expositions in the virology field, from intriguing or persuading scientists to stimulating novel research and future researchers.

## 3. A Fight for Supremacy: Peculiar Features of GVs and Their Interaction with Amoeba Hosts

Much of the biology and particularities of mimivirus interactions with their hosts were discovered during early investigations of GVs. In this regard, both imaging and genomic techniques (e.g., electron microscopy, atomic force microscopy, sequencing, etc.) were of pivotal importance in uncovering many peculiar features of mimiviruses. APMV, for example, was observed to attach to the host cell through glycoside interactions between the long fibrils and surface glycans, with such adhesion also occurring with other unrelated organisms (e.g., arthropods and fungi), potentially facilitating the dispersion of these viruses in the environment [70].

Once they reach their hosts, differently from most non-giant viruses, mimiviruses (and most of the described GVs) enter the host cell through phagocytosis [71]. This was initially observed by transmission electron microscopy (TEM) analysis and further corroborated by biological assays, especially in cells treated with phagocytosis and endocytosis inhibitors [72]. At the apex of the mimivirus capsid there is a starfish-shaped protein complex that acts as a seal for the stargate, until the phagosome’s internal environment promotes a new protein arrangement, unleashing the opening of the stargate and the release of the genome [73,74]. The acidification of the phagosome is suggested to be a factor that leads to capsid disassembly and membrane fusion [72,74,75].

**Figure 5:**
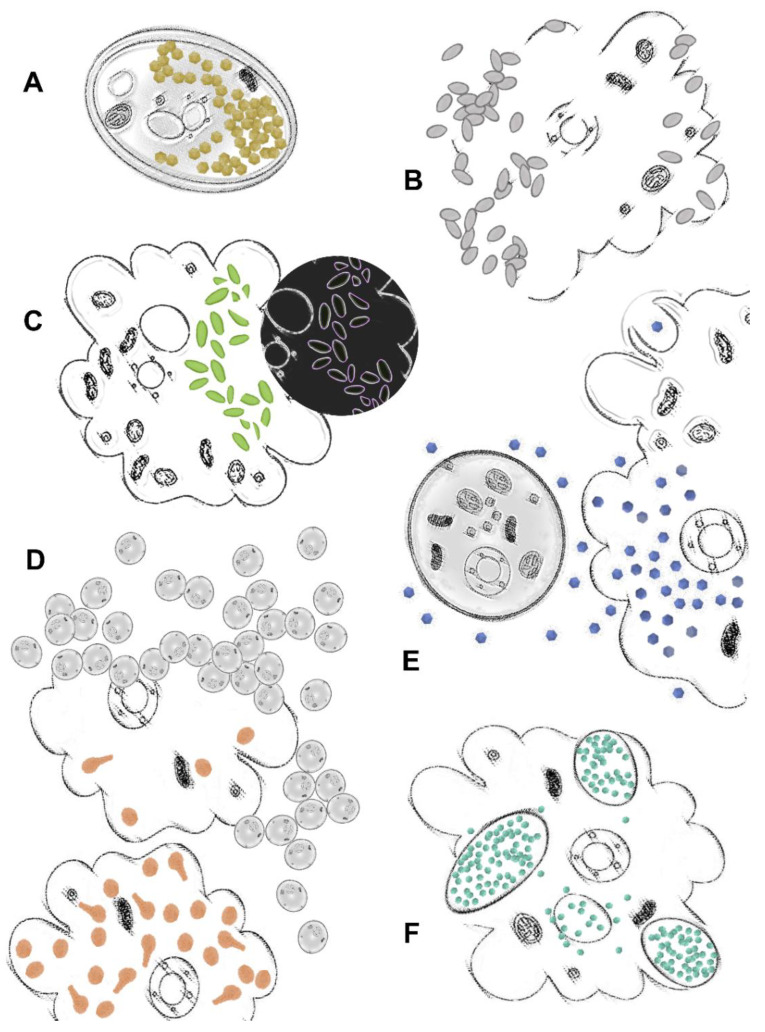
Unique features of giant viruses’ (GVs’) replication cycles unraveled in Brazil. (**A**) faustovirus dissemination is circumvented by amoebas with the enclosing of viral progeny inside the host’s cysts [26]; (**B**) orpheovirus particles are released from the host by exocytosis or cell lysis [64]; (**C**) cedratvirus particles’ morphogenesis follows a unique and complex sequential organization, including horseshoe and rectangular compartments, the incorporation of the second cork and thickening of the capsid well, and finally the formation of the ovoid-shaped virion [24]; (**D**) amoebas infected with tupanvirus are induced to aggregate with uninfected cells, forming giant host cell bunches [78]; (**E**) mimiviruses are able to infect amoebal trophozoites and prevent encystment, while cysts are resistant to infection [81]; (**F**) MsV are able to form giant vesicles with numerous viral particles derived from amoebal endoplasmic reticulum [84]. Amoeba images were generated from free vectors available online at Vecteezy: https://www.vecteezy.com (accessed on 22 September 2021).

In this regard, in 2011, the isolation of SMBV paved the way for further studies performed by other Brazilian research groups, enriching knowledge of mimiviruses’ structure and biology. These studies included analysis of different Gs particles using distinct imaging techniques, such as cryo-electron microscopy (cryo-EM) and tomography, as well as fluorescence microscopy [28,76]. In a structural study developed by Schrad et al. (2020), for example, the viral particles of SMBV, tupanvirus, antarctica virus and mimivirus M4 were used to investigate the process of genome release in mimivirus-like particles [77]. Taking this work as an example, the authors have corroborated the importance of conditions such as temperature and pH for the opening of the vertex in these GVs. Here, new additional information on the viral uncoating was settled, as liberation of the viral seed (extra membrane sac) and the complete release of the viral genome were both manifested by experiments using specific conditions of these GVs’ replication cycle (e.g., pH = 2 and/or 100 °C) [73]. Even though these conditions are non-biological, the authors suggest that they mimic GVs’ replication cycle steps. It may also suggest that during the replication cycle other factors may play a role in capsid opening. In the same study, during the steps of viral genomic release and by adopting different imaging techniques such as cryo-EM, cryo-electron tomography and scanning electron microscopy (SEM), the authors have observed the formation of pockets devoid of DNA within the nucleocapsids of these GVs. Likewise, the analyses led to the identification of a set of proteins released from capsids during the early stages of infection within this whole complex [73]. Looking into another study, with analyses involving SEM and TEM, the authors have observed that for different mimiviruses the density of the fibrils on the surface of their capsid was variable and that this could be acquired simultaneously to genome acquisition throughout the process of morphogenesis in the large viral factories [72]. These techniques were also important in revealing key aspects of the replication cycle of different giant viruses. As already mentioned above, an antiviral strategy was beautifully described in a TEM study showing faustovirus mariensis particles trapped inside the cysts of the *Vermamoeba vermiformis* host (Figure 5A) [26]. An in-depth description of the replication cycle of orpheovirus and cedratvirus was also established, mostly by imaging methods. For orpheovirus, we started to understand that viral exocytosis was as important as the cell lysis to the final step of this giant virus cycle (Figure 5B) [64]. For cedratvirus getuliensis, the contribution of these techniques has helped to describe a unique and complex sequential organization of the viral particle morphogenesis, including different steps of the formation of horseshoe and rectangular compartments, the incorporation of the second cork and thickening of the capsid well, and finally the formation of the ovoid-shaped virion (Figure 5C) [24]. Considering some intriguing observed features after the mimiviruses’ release, another interesting study was proposed by Oliveira et al. (2019) and observed an aggregation of released tupanvirus particles with uninfected amoeba, promoting viral dissemination by the formation of host cell bunches (Figure 5D) [78]. This study revealed that this amoebal-bunch formation is correlated with the mannose-binding protein (MBP) gene expression, either induced by tupanviruses or between amoebas, through interactions among their receptor, both factors that may be important for the optimization of this process [78]. However, when we talk about the genome of tupanviruses, we observe that a great number of their genes are not present in many other mimiviruses’ genomes [10], which may still hide some important information about these GVs’ cycle. In view of how life has evolved on Earth, the complexity of this genome has also recently been used as an argument to suggest that viruses come from an ancestral strategy of life. According to the authors, in the period comprising the First to the Last Universal Common Ancestor (FUCA to LUCA), an intermediate ancestral (Transitional-LUCA) may have been arisen as an undifferentiated subsystem resembling a virus-like structure, from which most of the currently known viruses came [79]. Besides, aside from the already mentioned unique structural tail, and the formation of bunches [78], tupanviruses exhibited for the first time a cytotoxic phenotype to non-host cells [10]. These intriguing aspects metaphorically resemble a constant fight for supremacy [80] and help unravel the evolutionary history of GVs.

In addition to these distinct characteristics, it is worth mentioning that, as expected, the host cell does not remain indifferent to mimivirus infection. The encystment process is understood as a mechanism used by *Acanthamoeba* populations to become protected against several kinds of stressful conditions, such as dehydration, lack of nutrients, UV light, and viral infections, including against mimiviruses [26,81,82]. As observed in a study developed by Boratto et al. (2015), mimivirus infection is hampered even if those amoebas are not yet morphologically encysted but had already received the stimulus to turn into their resistant form (Figure 5E) [81]. Nonetheless, if the stimulus to become a cyst is triggered before the infection, mimiviruses as APMV are able to evade this protective status of the *Acanthamoeba* cyst, by preventing the expression of an encystment-mediating subtilisin-like serine protease and thus proceeding with the infection (Figure 5E) [81]. These studies demonstrate how complex are processes involving GVs’ replication cycle and what an intricate interaction these viruses have with their amoebal hosts.

In addition to isolation studies already mentioned above, other works developed in Brazil have contributed to add knowledge of genomics and important relationships between marseilleviruses and their host. One of these studies has helped to bring light to pivotal processes in the replication cycle of marseilleviruses, specifically related to the viral entry and release. As extensively described in the literature, phagocytosis is a general route used by most GVs to enter amoebal cells [1,6,53,80]. This process is triggered only after recognition of particles larger than 500 nm [83]. However, viruses with particle sizes between 200 to 250 nm, as is the case for marseilleviruses, do not have the minimum size required to trigger this process [84].

By performing an in-depth investigation of the marseillevirus replication cycle and using a different set of virological assays (e.g., TEM, SEM, immunofluorescence, immunoblotting), Arantes et al. have shown that during marseillevirus assembly the viral particles are organized inside large vesicles (some reaching about 3 µm in size) which are originated from the endoplasmic reticulum of the infected cells (Figure 5F) [84]. After viral release, those particles are then ready to infect another cell by exploring the phagocytosis of these vesicles that contain dozens to thousands of viral particles in their interior. In addition, viral release also seems to occur by individual virions. In this case, marseilleviruses exploit the endocytosis route to enter the cell by a mechanism which is dependent on acidification [84].

The *Marseilleviridae* family is also well known for its genomic mosaicism, which consists of the ability to incorporate foreign genes from other organisms that have *Acanthamoeba* as a common host [6]. Genomic studies of several strains of marseillevirus showed the presence of an A-T-rich promoter motif (AAATATTT) that is associated with 55% of the viral genes and that is conserved among all lineages. In addition, biological assays showed that the alteration of the promoter sequence negatively impacts the genes’ transcription, showing a possible link of these sequences to the increased expression of some genes [85]. The presence of multiple copies of these motifs in the intergenic regions suggests that they may favor the fixation of newly acquired genes [85].

More recently, in 2020, analysis of the marseillevirus transcriptome revealed a temporal gene expression profile, indicating the existence of three categories: early, intermediate and late [86]. Genes belonging to different functional groups exhibited distinct expression levels throughout the infection cycle and marseillevirus infection causes significant changes in the host’s transcription machinery, downregulating many genes [86].

Finally, it is worth mentioning that much of the features above described for GVs and their hosts have an influence directly affected by the intracellular environment of the amoebas. This environment has already been seen as an ecological site that comprehends a number of different and phylogenetic distant microorganisms, which not only inhabit the same location but are also observed to be in a strong process of coevolution. Even if not genetically related, an important portion of the genomic signatures (described as “the total net response to selective pressures”) of the coevolving microorganisms are found to be incredibly conserved. This makes the intracellular environment of the amoebal host a sanctuary for interactions among several species of ecological and biomedical relevance [87].

## 4. Giant Viruses As a Tool to Update and Inspire: From the Research Fields to the Classroom

Since the known virosphere is notably anthropocentric, virology classes usually present viruses as pathogenic organisms, strongly associated with human diseases [3,88]. Instead of presenting these organisms as important tools of natural selection, ecological balance and the Earth’s biogeochemical cycles, the commonly used material for teaching virology leads to a biased misconception of viruses as strictly bad, generating a certain fear in the students [89]. Besides this, other problems are the high cost of ensuring biosafety for practical virology classes, and motivation and mastery of the subject by the teachers (especially at the elementary school level) [90]. A further point is that viruses are typically very abstract for students, mainly due to their size, which limits their visualization to schematic figures, illustrations, and electron microscopy images [88].

The expansion of the perception of the virosphere by the giant viruses has unleashed a new way of understanding and teaching virology. Due to their colossal particles, the size limitation has been considered obsolete, turning these viruses into excellent learning tools [88,91]. Therefore, GVs can be visualized by optical microscopy, like bacteria and fungi, which are traditionally presented to students through common microscopes. Moreover, since they infect free-living amoebae, they represent a safe and low-cost instrument for practical virology classes [88,91].

**Figure 6:**
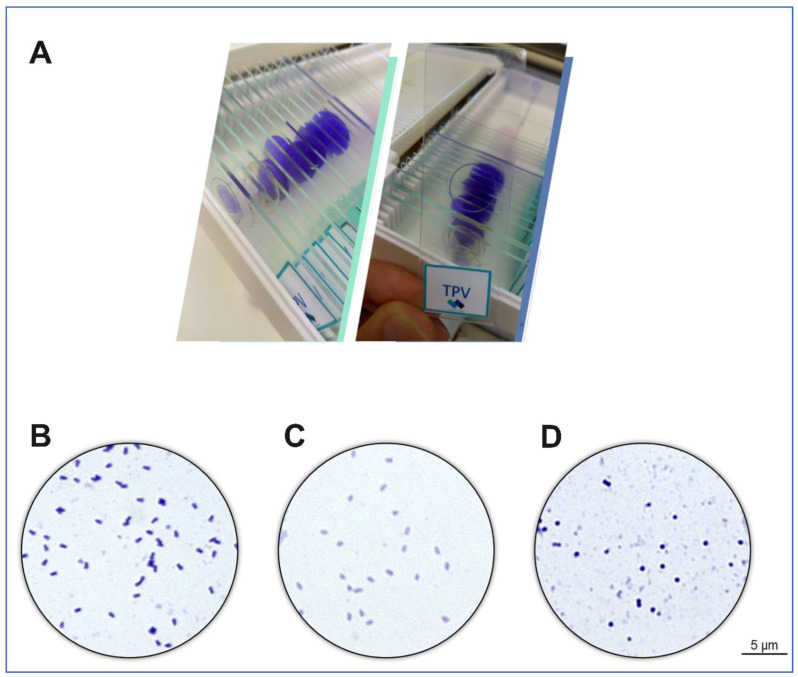
Optical microscopy images. (**A**) Source: Reference 91]. Visualization under 1000 times magnification of the stained purified particles of (**B**) tupanvirus, (**C**) cedratvirus and (**D**) Niemeyer virus, respectively.

In 2020, our group developed an educational kit to update the content typically taught in virology classes and align it to recent breakthroughs in virus research. Using slides, staining materials, viruses from the laboratory stock, and cell lineages, a microscope slides kit called “Virus Goes Viral” was created (Figure 6A) [91]. This allows students to observe giant viruses’ particles (Figure 6B–D), viral factories, and different lysis plaques in important viruses that infect animals [91]. As basic research regarding GVs in the Brazilian territory may enrich the knowledge of these microorganisms in the virology field, this kit may also aim to foster an inspiring learning environment, as well as ignite more interest in these fascinating organisms in the future.

## 5. Conclusions

The serendipitous discovery of APMV in 2003 changed the concept of viruses and expanded the limits of the virosphere [1]. Over the last two decades, different groups of large and giant viruses of amoebae have been described throughout the world, revealing many unusual particles’ shapes and genome length and content. Culture-independent studies have proved the ubiquity and the astonishing diversity of giant viruses on Earth [18,68]. Now, we must go deeper into the characterization of these viruses, by using different isolates as models. Several new viruses isolated from distinct viral families (e.g., *Mimiviridae* and *Marseilleviridae*) are now under in-depth investigation to better understand their biology and evolution, and these outstanding discoveries may even change our perception of life itself (e.g., *Mimiviridae* as a new branch derived from a population that gave origin to the modern Eukarya [20]).

Brazil is the fifth largest country in terms of territory and harbors different biomes, making the country an important global hub of tropical biodiversity. Over the past 10 years, the diversity of amoebal viruses in Brazilian environments has been uncovered, with hundreds of isolates belonging to distinct groups, including members of *Mimiviridae*, *Marseilleviridae*, Pandoraviridae, Pithoviridae, Faustoviridae, *Lavidaviridae*, among others, as discussed in previous sections. Interestingly, the most complex giant viruses described to date were isolated from two distinct areas in Brazil [10]. Furthermore, the recent discovery of the small mysterious yaravirus in the country highlighted the importance of continuing the search for new isolates, which could reveal completely new entities on Earth [27]. Brazilian groups, working alongside other experts in the field, have contributed to uncovering this unusual and exciting side of the virosphere. The study of amoebal viruses has already changed our perception of basic virology. Furthermore, giant viruses have recently been proposed as tools to improve virology learning at different educational levels [91]. Surely, other potential applications for these viruses are waiting to be revealed as new data emerges. This is an open field for remarkable discoveries, and we can expect great innovations as new amoebal viruses are isolated and characterized. In this context, the preservation of Brazilian biomes is a sine qua non condition, not only for the discovery of novel biological entities (including giant viruses), but also because of climatic, philosophical, political and economic reasons. Finally, we would like to reinforce that, although this review is a celebration of the 10th anniversary of giant virus studies in Brazilian biomes, we do not support any kind of excessive scientific nationalism. We are aware that studies of giant viruses in Brazil represent a modest contribution to the giant viruses’ universe. This field has been constructed by remarkable worldwide research groups, and we are very grateful for their efforts and inspiring work.

## Figures and Tables

**Table 1:** General features of Brazilian giant viruses with complete sequenced-genomes.

Group of Virus	Virus	Type of Sample	Location (Year of Isolation)	Genome Size (bp)	ORFs	ORFans	GC %	Reference
								
	Samba virus	Fresh water	Negro River (2011)	1,181,380	971	0	27	Campos et al., 2014
	Amazonia virus	Fresh water	Negro River (2011)	1,179,119	979	1 (0.1%)	27	Assis et al., 2015
*Mimiviridae*	Kroon virus	Urban lake water	Lagoa Santa city (2012)	1,221,932	944	3 (0.3%)	27	Assis et al., 2015
(lineage A mimivirus)	Oyster virus	Oysters	Santa Catarina state (2013)	1,200,220	948	1 (0.1%)	27	Assis et al., 2015
	Niemeyer virus	Urban lake water	Pampulha Lagoon (2011)	1,299,140	1003	0	28	Boratto et al., 2015
								
								
*Mimiviridae*	Borely moumouvirus	Fresh Water	Serra do Cipó (2018)	1,038,187	947	3 (0.3%)	25.2	Silva et al., 2020
(lineage B mimivirus)								
								
								
*Mimiviridae*	Mimivirus gilmour	Urban lake water	Pampulha Lagoon (2014)	1,258,663	1135	28 (2.4%)	26	Assis et al., 2017
(lineage C mimivirus)	Mimivirus golden	Golden mussels	Guaíba Lake (2014)	1,248,960	1127	19 (1.6%)	26	Assis et al., 2017
								
								
*Mimiviridae*	Tupanvirus deep ocean	Deep Ocean sediments	Campos dos Goytacazes city (2018)	1,439,508	1276	378 (29.6%)	28	Abrahão et al., 2018
	Tupanvirus soda lake	Soda Lake	Nhecolândia, Pantanal biome (2018)	1,516,267	1359	375 (27.6%)	28	Abrahão et al., 2018
								
								
*Marseilleviridae*	Brazilian marseillevirus	Sewage	Pampulha Lagoon (2014)	362,276	491	29 (5.9%)	43.3	Dornas et al., 2016
	Golden marseillevirus	Golden mussels	Guaíba Lake (2014)	360,610	483	43 (8.9%)	43.1	Santos et al., 2016
								
								
Cedratviruses	Brazilian cedratvirus	Water supplemented with biofloc	Belo Horizonte city (2018)	460,038	533	11 (2.1%)	42.9	Rodrigues et al., 2018
								
								
Faustovirus	Faustovirus mariensis	Urban lake water	Pampulha Lagoon (2019)	466,080	483	0	36	Borges et al., 2019
								
								
Yaravirus	Yaravirus brasiliensis	Muddy water	Pampulha Lagoon (2020)	44,924	74	68 (91.9%)	57.9	Boratto et al., 2020
								

## Data Availability

Genomic data can be found at genbank (https://www.ncbi.nlm.nih.gov/genbank/, accessed on 11 January 2022).
